# Rhinologic outcome of endoscopic transnasal-transsphenoidal pituitary surgery: an institutional series, systematic review, and meta-analysis

**DOI:** 10.1007/s00405-023-07934-w

**Published:** 2023-03-29

**Authors:** Nathalie A. Gstrein, Sebastian Zwicky, Carlo Serra, Michael Hugelshofer, Luca Regli, Michael B. Soyka, David Holzmann, Christian M. Meerwein

**Affiliations:** 1 Department of Otorhinolaryngology, Head and Neck Surgery, University Hospital Zurich, University of Zurich, Frauenklinikstrasse 24, 8091 Zurich, Switzerland; 2 Department of Neurosurgery, Clinical Neuroscience Center, University Hospital Zurich and University of Zurich, Zurich, Switzerland

**Keywords:** Endoscopic, Transnasal-transsphenoidal, Pituitary, Nasal, Function, Rhinologic

## Abstract

**Purpose:**

We aimed to summarize the available data on the objective rhinologic outcome after endoscopic transnasal-transsphenoidal (ETT) surgery.

**Methods:**

Retrospective study on a consecutive cohort of treatment-naïve patients undergoing ETT pituitary gland surgery. Additionally, a systematic review and meta-analysis with focus on the rhinologic outcome, including postoperative smell function was performed.

**Results:**

The institutional series incorporated 168 patients. A concomitant endoscopic septoplasty was performed in 29/168 patients (17.3%). A nasoseptal flap was used for reconstruction of large skull-base defects or high-flow CSF leaks in 4/168 (2.4%) patients. Early postoperative rhinologic complications (< 4 weeks) included epistaxis (3%), acute rhinosinusitis (1.2%) and late postoperative complications (≥ 8 weeks) comprised prolonged crusting (15.6%), symptomatic synechiae (11.9%) and septal perforation (0.6%). Postoperative smell function was not impaired (Fisher’s exact test, *p* = 1.0). The systematic review included 19 studies on 1533 patients with a median postoperative epistaxis rate of 1.4% (IQR 1.0–2.2), a postoperative acute rhinosinusitis rate of 2.3% (IQR 2.1–3.0), a postoperative synechiae rate of 7.5% (IQR 1.8–19.1) and a postoperative septal perforation rate of 2.2% (IQR 0.5–5.4). Seven studies including a total of 206 patients reported adequate outcome measures for smell function before and after ETT surgery. Only 2/7 studies reported an impairment of smell function postoperatively, especially in patients with nasoseptal flap harvesting.

**Conclusion:**

Early and late postoperative rhinologic complication rates after ETT surgery for pituitary lesions seem to be low. A thorough evaluation of smell function, in particular in patients at risk for nasoseptal flap harvesting, may be an important factor in optimal postoperative care.

**Supplementary Information:**

The online version contains supplementary material available at 10.1007/s00405-023-07934-w.

## Introduction

The introduction of the endoscopic transnasal-transsphenoidal (ETT) approach has significantly changed the surgical concept of treating pituitary gland lesions. While historically both transcranial and sublabial approaches with the use of a microscope have been utilised, the introduction of a transnasal endoscopic route led to a paradigm shift [1–5]. The formation of a wide transnasal corridor through the patient’s nostril not only improves surgical exposure, but also accommodates surgical instruments and allows the harvesting of mucosal flaps for reconstruction [6, 7]. Additionally, compared to traditional surgical concepts, the ETT approach is associated with reduced morbidity and aims to preserve nasal function and maintain patients’ quality of life (QoL) [7]. When comparing traditional microscopically guided approaches to the ETT approach, based on disease-specific QoL questionnaires, long-term nasal function was superior and the need for sinonasal treatment and medication was reduced in the ETT group [2]. More extended endonasal approaches, such as the extended transnasal-transsphenoidal approach to the skull base, which usually includes middle turbinate removal, seem to negatively impact the patient’s olfaction and mucociliary clearance at 3 months [8].

In the early postoperative phase after ETT surgery, epistaxis and acute rhinosinusitis may occur [7]. Late complications include extensive nasal crusting, septal perforation, and synechiae [7]. Although many series have not reported an overall decrease in smell function after ETT pituitary surgery, olfactory impairment should be considered, particularly on the donor side of ETT patients undergoing skull base reconstruction with a nasoseptal flap [7, 9]. Based on these findings, monorhinal smell testing before surgery should be considered, possibly preventing smell impairment in patients with pre-existing single-sided olfactory loss [9]. However, at many centres, a standardised assessment of both objective and subjective preoperative and postoperative nasal function after ETT surgery is not routinely implemented in the treatment pathway for pituitary gland lesions. Furthermore, as it was outlined above, various retrospective single-centre studies have reported rhinologic outcomes of ETT surgery [7–9]. However, both, the reported study endpoints and the presented outcomes are heterogeneous, which hinders clear interpretation. With this study, which incorporates our on institutional data, we aimed to summarise the available data on this topic, particularly focusing on the olfactory outcome of these patients.

## Methods

### Study design

This study received approval from the ethical committee of the canton of Zurich, Switzerland (approval number: 2021-00926). Patients with documented denial to contribute personal health-related data to research were not included. We retrospectively reviewed a consecutive cohort of adult patients treated for pituitary gland lesions at the University Hospital Zurich (Switzerland). The study duration was set from January 2013 to October 2022, as we systematically changed from the transnasal-microscopic approach to the ETT approach at the beginning of January 2013. Only primary cases (no revision cases) undergoing an ETT approach were included. Extended transnasal-transsphenoidal approaches (e.g. transplanum approach) were excluded. A nasoseptal flap was harvested in cases with an intraoperative high-flow cerebrospinal fluid (CSF) leak and/or large skull-base defects. A high-flow leak was defined as large dural skull base defect and basal cisterns or ventricular opening, while small dural defect with moderate CSF leak was defined as low flow leak [10]. A single-shot of intravenous Cefuroxime was administered for antibiotic prophylaxis. The surgical technique consisted of an endoscopic unilateral sphenoidectomy, removal of the base of the vomer bone and existing intersphenoidal septum and finally, exposure of both sphenoids, including the medial and lateral optico-carotid recesses. A unilateral sphenoethmoidectomy was performed to increase visualisation and instrument manoeuvrability in selected cases. The superior turbinate and the cranial septal mucosa were preserved, to spare the olfactory fibres. If necessary, an endoscopic septoplasty with an incision just anteriorly of the deviated parts of the septum was conducted to improve accessibility, widen the surgical corridor and/or to prevent formation of synechia in the postoperative course. In all cases, a mononostril approach with 0° endoscopes (Karl Storz, Germany) was performed. After exposure of the sellae, the anterior wall was widely opened, followed by a X-shaped durotomy. Intradural adenoma removal was achieved using standard techniques. In selected cases, the endoscopic assisted “diving technique” was applied [11]. In the majority of patients, intraoperative high-field 3-T-MRI was used to visualize extent of resection [12]. Postoperative sphenoid packing consisted of Floseal haemostatic matrix (Baxter Advanced Surgery, US), Spongostan Absorbable Haemostatic Gelatin Sponge (Ethicon, Germany) and standard foam-based nasal packing (Netcell, Novimed, Switzerland) in one or both nasal cavities. Nasal packing was removed on the second postoperative day. Postoperative nasal care consisted of daily nasal rinsing with isotonic solutions. A first rhinologic appointment was scheduled 2 weeks postoperatively to remove crusts and debris. The majority of the procedures were performed in close collaboration between neurosurgeons (L.R., C.S., M.H.) and rhinologists (D.H., M.B.S.).

### Patient characteristics, surgical protocols, outcome measures, and follow-up

The following patient data and tumour data were collected: age, gender, date of surgery, macroadenoma (> 10 mm) vs. microadenoma (≤ 10 mm), and hormone secreting vs. non-hormone secreting. Preoperative rhinologic findings were categorised as (1) documented relevant septal deviation, (2) pre-existing septal perforation, and (3) long-distance synechiae. Objective postoperative rhinologic outcomes were categorised as early postoperative complications (haemorrhage with conservative or surgical treatment, acute rhinosinusitis with documented treatment, CSF leak, as documented by beta-transferrin testing) versus late-postoperative complications (prolonged crusting (> 8 weeks), long-distance synechiae, septal perforation). An early postoperative complication was defined to occur within 4 weeks after surgery, whereas a late complication occurred at ≥ 4 weeks, postoperatively. Acute rhinosinusitis was defined as a combination of patient-related symptoms (pain, pressure, nasal discharge) and endoscopic findings (purulent secretion, oedematous nasal mucosa). The location of the synechiae was categorized into (1) nasal septum—inferior turbinate, (2) nasal septum—middle turbinate, (3) superior turbinate—sphenoidotomy and (4) complex synechiae involving the nasal septum—inferior turbinate—middle turbinate. A long distance synechia was defined as every synechia, which was more extended than just a punctiform contact. Prolonged crusting was defined as: (1) symptomatic (nasal obstruction, nasal discharge), (2) detected by nasal endoscopy, (3) requirement of regular endoscopic cleaning, (4) present for more than 8 weeks. The pre- and postoperative olfactory function, as measured by an odour-identification test (Sniffing Sticks Smell test^®^; 0–12 points), was recorded. In accordance with Hummel et al., Sniffin-Sticks” test values of ≥ 10 points were considered normosmia, 7–9 points were hyposmia, and ≤ 6 were anosmia [13]. Postoperatively, as part of our treatment pathway, patients underwent a rhinologic assessment, including a nasal endoscopy with a particular focus on endonasal complications and a bilateral odour-identification test within 8–12 weeks after surgery. Monorhinal smell testing (as assessed by the Sniffing Sticks test for the right and left side separately) was performed if a nasoseptal flap was harvested. In the case of any rhinologic complications, follow-up was extended as needed.

### Systematic review

**Fig. 1:**
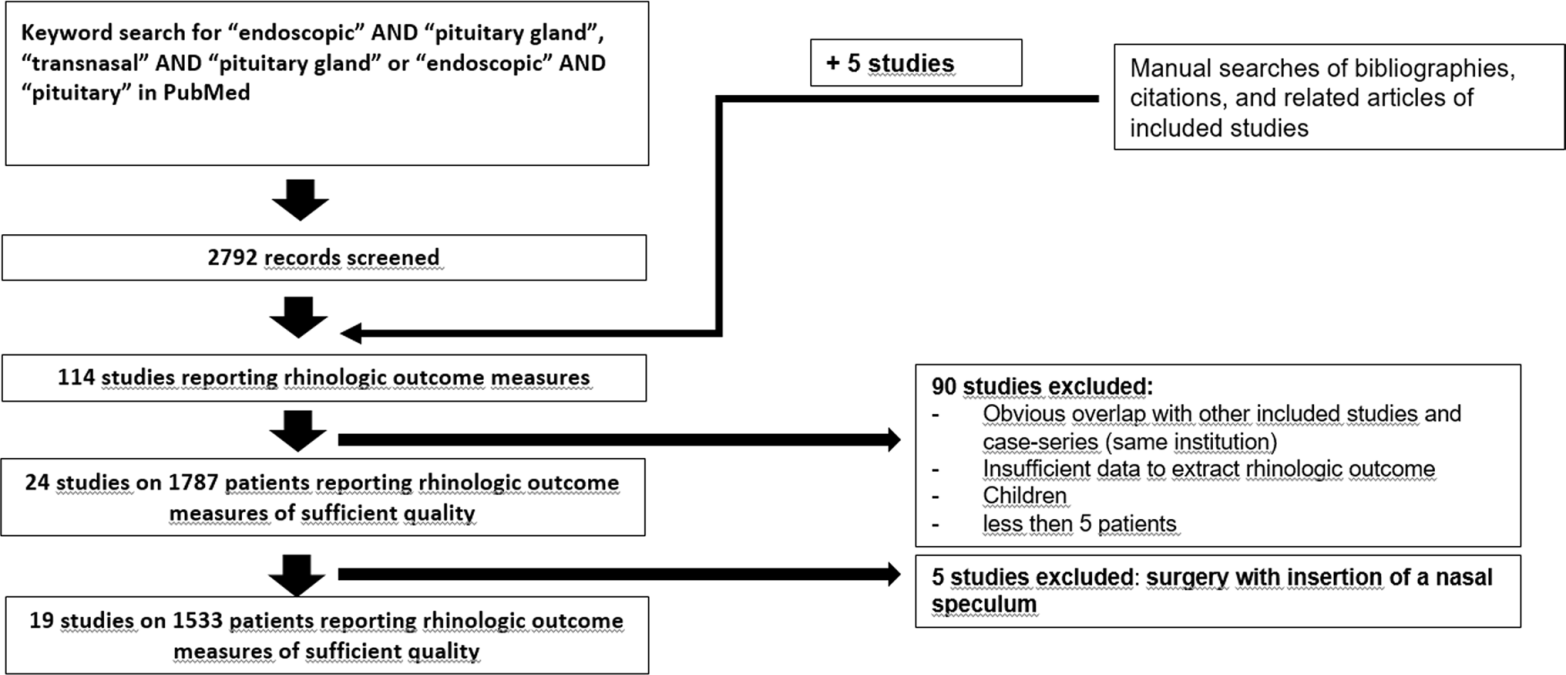
Details on the search strategy

A systematic review and meta-analysis were performed and reported according to the Preferred Reporting Items for Systematic Reviews and Meta-Analyses (PRISMA) guidelines. The data was extracted from PubMed using keyword searches containing “endoscopic” AND “pituitary gland”, “transnasal” AND “pituitary gland” or “endoscopic” AND “pituitary”. Only original English language articles on adult human subjects with a minimum of five reported patients were considered. The search was performed on 11 September 2022, and included studies from January 1992 to July 2022. After removing duplicates, a total of 2792 full texts were screened. The complete systematic review workflow is outlined in Fig. 1. Included studies were required to explicitly focus on the rhinologic outcome of ETT pituitary gland surgery. The rhinologic outcome was categorised according to objective-outcome measures: epistaxis/postoperative haemorrhage, crusting, septal perforation, synechiae, postoperative acute rhinosinusitis and smell impairment. At least two outcomes had to be reported for a study to qualify for the systematic review. Because this systematic review provides a synthesis of all available evidence regarding rhinologic outcomes, the quality and heterogeneity of the studies were assessed through a descriptive analysis instead of an *I*-squared statistics (*I*^2^) report. The aim of performing a systematic review was to provide a thorough compilation of the existing data on the rhinologic outcomes of ETT surgery.

### Variables and statistical analysis

Data are either presented as median and interquartile range (IQR) or as mean ± standard deviation (SD), depending on the normality of data distribution. Differences among pre- and postoperative smell function was calculated using contingency tables and Fisher’s exact test. The end of follow-up was July 2022. A *p* value less than 0.05 indicated significance. We used SPSS version 22 (IBM, Armonk, NY) for the statistical analysis.

## Results

### Institutional case series (University Hospital of Zurich, Switzerland)

In the period between January 2013 and October 2022, 320 patients underwent ETT surgery for pituitary gland tumours. Of those, 168 met the inclusion criteria. Table 1 provides details on patients’ baseline characteristics and definitive histopathological workup, including the hormonal status of the removed adenomas. Preoperative endoscopic findings were documented in 103/165 patients (62.4%) and revealed a relevant septal deviation in 67/103 patients (65%), a pre-existing septal perforation in 1/103 patient (0.97%) after septoplasty and pre-existing synechiae in 2/103 patients (1.9%) after previous functional endoscopic sinus surgery. All patients underwent ETT using a mononostril approach. A concomitant endoscopic septoplasty was performed in 29/168 patients (17.3%). A nasoseptal flap was used for reconstruction of large skull-base defects and/or high-flow CSF leak in 3/168 (1.8%) patients with macroadenomas and in and in one patient with a tuberculum sellae meningioma. Details on complication rates in the early and late postoperative course are presented in Table 2.

**Table 1:** Baseline patient and pituitary gland tumour characteristics

Characteristics	
Number of patients (*n*)	168
Gender (*n*, %)	
Female	82
Male	86
Age at diagnosis (median, 1.–3. IQR)	57 (42–68 IQR)
Final histopathological diagnosis (n, %)	
Adenoma	165/168 (98.2%)
Macroadenoma (> 10 mm)	158/165 (95.8%)
Microadenoma (< 10 mm)	7/165 (4.2%)
Sella meningioma	1/168 (0.6%)
Hemangiopericytoma	1/168 (0.6%)
Ruptured and infected Rathke cyst	1/168 (0.6%)
Hormone-status adenomas (*n*, %)	
Hormone-secreting	65/165 (39.4%)
Non-hormone-secreting	100/165 (60.6%)

**Table 2:** Early and late postoperative outcomes including smell function for our institutional series

Early postoperative complications (*n* = 168)	
Postoperative epistaxis, conservative treatment	2/168 (1.2%)
Postoperative epistaxis, sphenopalatine artery ligation	3/168 (1.8%)
Postoperative acute rhinosinusitis, treated with antibiotics	2/168 (1.2%)
Postoperative CSF leak	1/168 (0.6%)
Late postoperative complications (*n* = 160)	
Prolonged crusting	25/160 (15.6%)
Long-distance synechiae	19/160 (11.9%)
Septal perforation	1/160 (0.6%)
Preoperative smell function (*n* = 132)	
Normosmic	106/132 (80.3%)
Hyposmic	20/132 (15.2%)
Anosmic	6/132 (4.5%)
Postoperative smell function (*n* = 148)	
Normosmic	119/148 (80.4%)
Hyposmic	23/148 (15.5%)
Anosmic	6/148 (4.1%)

*CSF* cerebrospinal fluid

Most patients were seen for a specific rhinologic assessment after 2 weeks and after 8–12 weeks postoperatively (145/168, 86.3%). The median follow-up time was 4 months (IQR 3–8). Extensive synechiae were observed in 19/160 patients (11.9%). Synechiae involving the nasal septum and inferior turbinate were present in 6/19 patients, those involving the septum and the middle turbinate were seen in 6/19 patients, those between the superior turbinate and sphenoidotomy were seen in 1/19 patients and complex synechiae involving the septum, and inferior and middle turbinates were observed in 6/19 patients. Larger and symptomatic synechiae were incised under local anaesthesia, whenever they were detected during the rhinologic follow-up. It is noteworthy that no patient required a surgical intervention under general anaesthesia for symptomatic synechiae.

As seen in Table 3, most of the patients underwent preoperative (132/168 patients, 78.6%) and postoperative (148/168 patients, 88.1%) assessments of smell function between 8 and 12 weeks after surgery. When we compared the pre- and postoperative smell functions (normal smell function vs. impaired smell function), we did not find a difference (Fisher’s exact test, *p* = 1.0). A subanalysis of the four patients with nasoseptal flaps revealed a postoperative normosmia in 2/4 patients and a persisting hyposmia in 2/4 patients.

**Table 3:** Summary of olfactory outcome before vs. after ETT for studies matching the inclusion criteria for ETT surgery

	Objective test	Result	Comment
Hart et al. [37]	UPSIT (0–40 points)	Preop.: 31.7 ± 4.5 Postop. 1 month: 31.9 ± 4.0 Postop. 3 months: 32.9 ± 4.0	Pre- to postoperative function not impaired No nasoseptal flap
Rotenberg et al. (15)	UPSIT (0–40 points)	Preop.37.2 (SD not reported) Postop. 6 months: 30.8 (SD not reported)	Postoperative function impaired Nasoseptal flap in all patients No monorhinal testing
Kahilogullari et al. (38)	Smell diskettes (0–8 points)	Preop.: all normosmic Postop. 1 month: 2/25 hyposmic Postop. 6 months: 0/25 hyposmic	Pre- to postoperative function not impaired No nasoseptal flap
Alobid et al. [8]	Barcelona smell test (detection, identification, forced choice)	Preop.: 95.2 ± 9.5% detection, 23.1 ± 22.3% identification, 55.2 ± 26.6% forced choice Postop. 3 months: 94.2 ± 9.9% detection, 20.9 ± 20.2% identification, 61.5 ± 19.9% forced choice	Pre- to postoperative function not impaired EEA similar to TTEA Nasoseptal flap in all patients No monorhinal testing
Schreiber et al. [23]	UPSIT (0–40 points)	Preop.: 28.3 ± 6.7 Postop. 6 months: 27.5 ± 6.9	Pre- to postoperative function not impaired Nasoseptal flap in 5/29 patients
Tam et al. [39]	UPSIT (0–40 points)	Preop.: all normosmic, 34–40 Postop. with NSF at 6 months: 30.7 ± 1.77 Postop. without NSF at 6 months: 33.9 ± 1.45	Postoperative function impaired in both groups, NSF patients revealed greater loss of smell function
Sowerby et al. [40]	UPSIT (0–40 points)	Preop.: 34.8 ± 2.3 Postop. 3 months: 35.1 ± 3.0	Pre- to postoperative function not impaired Nasoseptal flap in patients in 4/22 patients

*EEA* endoscopic endonasal approach, *ETT* endoscopic transnasal-transsphenoidal, *NSF* nasoseptal flap, *TTEA* transnasal transsphenoidal endoscopic approach, *UPSIT* University of Pennsylvania Smell Identification Test

### Systematic review and meta-analysis

**Fig. 2:**
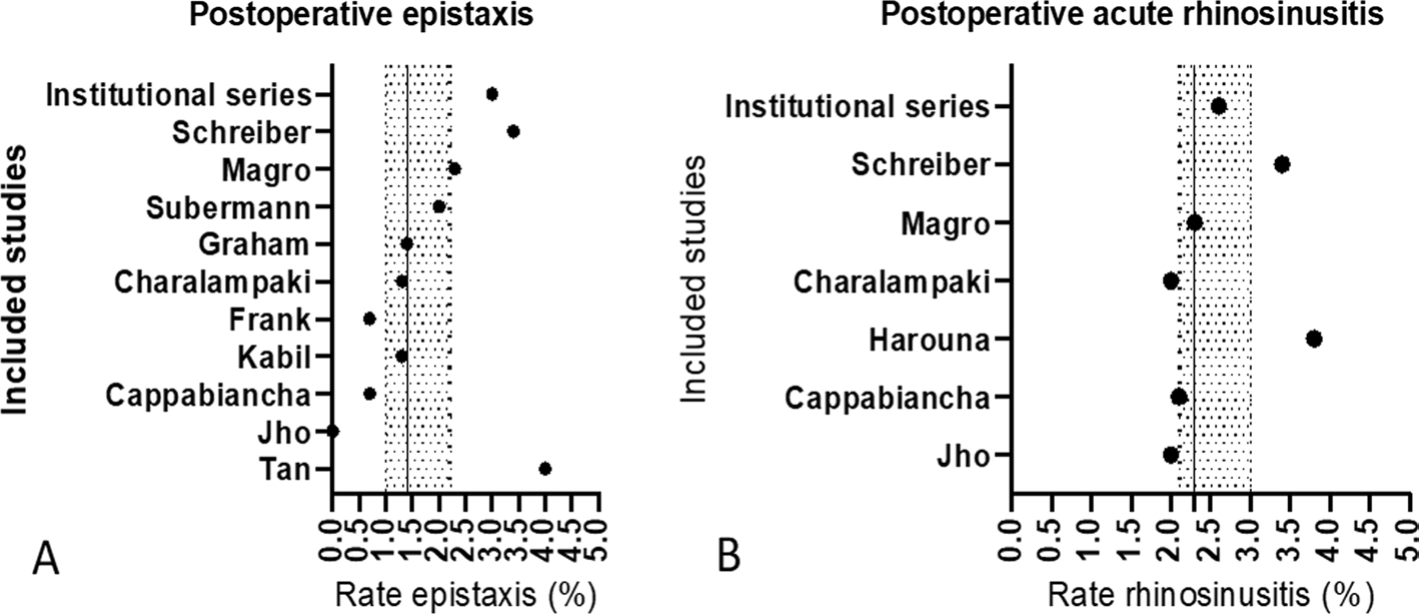
Early postoperative complication rates for all included studies matching the inclusion criteria for the meta-analysis (including our institutional series). Vertical line indicates median finding of all included studies (hatched area 1.–3. IQR). Median postoperative epistaxis rate (%, IQR): 1.4 (IQR 1.0–2.2). Median postoperative acute rhinosinusitis rate (%, IQR): 2.3 (IQR 2.1–3.0). IQR; interquartile range

**Fig. 3:**
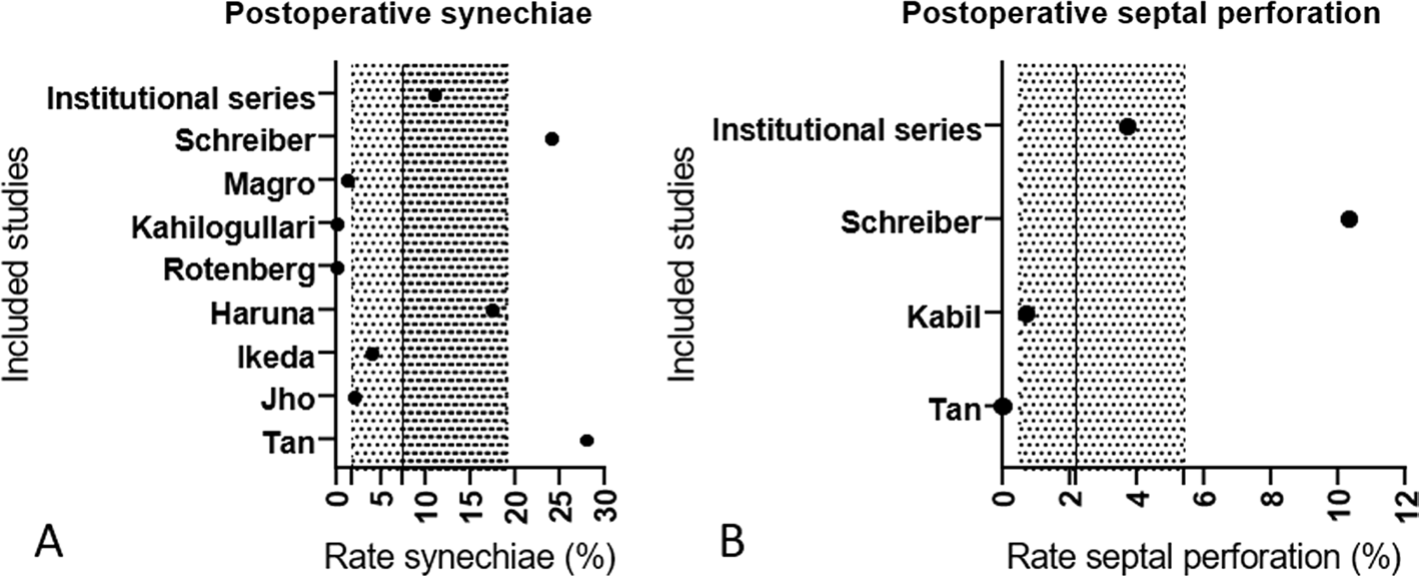
Late postoperative complication rates for all included studies matching the inclusion criteria for the meta-analysis (including our institutional series). Vertical line indicates median of all included studies (hatched area 1.–3. IQR IQR). Median postoperative synechiae rate (%, IQR): 7.5 (IQR 1.8–19.1). Median postoperative septal perforation rate (%, IQR): 2.2 (IQR 0.5–5.4). IQR; interquartile range

Our search yielded a total of 2,792 articles (Fig. 1). After screening the abstracts, we identified 114 full-text articles reporting outcome measures on transnasal endoscopic pituitary gland surgery. A total of 24 single- or multi-institutional studies published between 1995 and 2020 provided appropriate rhinologic outcomes. However, five studies were excluded, because, even though the surgical procedures were performed endoscopically, a nasal speculum was inserted. Finally, 19 studies from eight countries (United States of America, United Kingdom, Australia, Turkey, France, Italy, Germany, and Spain) were included in the analysis. We pooled the data of a total of 1533 patients. Supplemental Table 1 displays information on the rhinologic outcome per included study. Figure 2 provides detailed information on early and Fig. 3 on late postoperative complications, including our own data. A total of eleven studies (including our own institutional cohort) reported details on postoperative epistaxis and seven studies on postoperative acute rhinosinusitis rates: median postoperative epistaxis rate 1.4% (IQR 1.0–2.2); median postoperative acute rhinosinusitis rate 2.% (IQR 2.1–3.0). With regard to late postoperative complications, 9 studies providing information on postoperative synechiae and only four studies providing information on postoperative septal perforation were included: median postoperative synechiae rate 7.5% (IQR 1.8–19.1), median postoperative septal perforation rate 2.2% (IQR 0.5–5.4. Only three studies reported extensive postoperative crusting, with rates between 0 and 18.9% (mean 9.0%, ± 9.5) [14–16].

## Discussion

### Main findings

The incidence of early and late postoperative complications in primary ETT pituitary surgery is low. Pre- and postoperative olfactory function is comparable, as long as no nasoseptal flap is harvested. Assessment of smell function before and after surgery, as well as regular postoperative rhinologic follow-up examinations may be important factors in optimal postoperative patient care.

The endoscopic approach to the pituitary gland is considered minimally invasive compared to the microscopic technique, because neither a sublabial approach, transseptal incision or speculum insertion is required [17]. This translates into a reduced overall complication rate, operating room time, hospital stay and patient discomfort [17]. With particular focus on rhinologic complications, White et al. found a higher rate of postoperative epistaxis and septal deviation in the microscopic group [18]. In addition, higher rates of postoperative acute rhinosinusitis and synechiae were reported in the literature [19, 20]. Regarding postoperative smell function, some authors reported a more favourable outcome for the endoscopic approach [20, 21], while others could not confirm these findings, on the assertion that protecting the nasal mucosa is key, regardless of the approach [22].

Consistent with previous reports, the retrospective analysis of our institutional cohort revealed low rates of early and late postoperative complications. In the early postoperative phase, the predominant complication was bleeding from the septal branch of the sphenopalatine artery, which occurred in 3.0% of patients. In the literature, reported rates of postoperative epistaxis after ETT surgery varied between 0 and 4% [15, 23–32]. However, details on the origin of the postoperative epistaxis and treatment concepts were not provided in those studies. The five epistaxis episodes in our cohort consisted of two mild haemorrhages, which were managed conservatively with absorbable haemostatic gelatine sponge packing (Spongostan TM). However, in three patients we opted for surgical haemostasis under general anaesthesia with ligation of the septal branch of the sphenopalatine artery in two patients and coagulation of the sphenopalatine artery at the level of the sphenopalatine foramen in one patient due to diffuse bleeding. Thompson et al. even reported two patients with intrasellar haemorrhage after ETT surgery, which lead to temporary visual field defects [33]. The course of the sphenopalatine artery and its septal branches has been described in detail [34, 35]. According to Griffiths et al., the septal branches, also forming the arterial pedicle for the nasoseptal flap, run approximately 9 mm inferior to the sphenoid sinus ostium, allowing a safety margin to prevent transection [36]. At the minimum, one side of the posterior nasal septal artery should be preserved to maintain the possibility of harvesting a secondary nasoseptal flap; however, we opted for a low threshold, exploring the postoperative situs under general anaesthesia to achieve surgical haemostasis in cases of early postoperative bleeding [33].

The rate of postoperative acute rhinosinusitis has been reported to be between 2.0 and 3.8% (institutional cohort: 1.2%). This is most likely caused by stenosis of the sphenoidotomy, in which mucus retention combined with absorbable haemostatic packings cause stasis and consequential secondary infection. Therefore, certain authors claim that the entire mucosa of the sphenoid should be removed, to gain not only maximal exposure, but also minimal risk of postoperative infection. However, data supporting this manoeuvre is still lacking. Furthermore, a thorough postoperative care, with regular moisturising and rinsing, as well as endoscopic cleaning may prevent scarring and shrinking of the sphenoidotomy [7]. In our series, all postoperative sinus infections were managed with intensive local care and systemic antibiotics.

The late complications in our series were similar to the findings in the systematic review. Usually, we opt for a mononostril ETT approach, with the contralateral mucosa almost completely preserved. Given that we included only primary cases and tumours limited to the pituitary gland, the rate of septal perforations needs to be interpreted with caution, since a nasoseptal flap was only harvested in 4/168 (2.4%) patients. A postoperative septal perforation was seen in only one patient (0.6%) with a past medical history of septoplasty. In this case, most of the cartilaginous septum had been resected, leaving a very thin mucosa on both sides. In an Italian series on ETT surgery, Schreiber et al. reported that all septal perforations occurred in the area where the mucosa had been incised with monopolar electrocautery to harvest a nasoseptal flap or to create a corridor for transseptal sphenoidotomy [7]. In line with this observation, Kim et al. claimed that electrocautery causes more damage to the mucosa than the cold steel technique [37]. Long-distance synechiae were observed in 11.9% of patients, but remained asymptomatic in all patients, obviating the need for surgical intervention. In particular, avoiding speculum insertion may have been an important factor since the reported rates of microscopic synechiae were up to 38% [20].

Only seven studies involving a total of 206 patients reported adequate outcome measures for smell function before and after ETT [8, 16, 32, 38–41] (Table 3). Many studies were excluded, because the surgical procedures were performed endoscopically with an inserted speculum or because the reported outcome was heterogeneous and did not specify whether a microscopic or an endoscopic technique was applied [42–45]. In addition, many studies have relied on self-reported olfactory impairment, which is clearly inadequate [15, 30, 31]. Overall, only one group reported a significant difference in smell function [16]. In their series of 17 patients, all of whom had nasoseptal flaps, Rotenberg et al. reported an impairment in olfactory function at 6 months postoperatively [16]. In a randomised controlled trial on ETT surgery with and without nasoseptal flaps, the same group found an impairment in smell function (pre- vs. postoperatively) in both groups at 6 months, with a greater loss in the nasoseptal flap group [40]. In contrast, all other series, including our own, revealed preserved postoperative olfactory function. Despite the report of Sowerby et al. who did not find impairments of smell function in their subgroup of patients with nasoseptal flaps [41], a thorough analysis of olfactory dysfunction after ETT surgery imperatively requires information on the rate of harvested nasoseptal flaps. As demonstrated in our series, olfactory impairment on the donor side is encountered frequently and justifies monorhinal smell testing before surgery, to prevent possible bilateral smell impairment in patients with pre-existing single-sided olfactory loss [9]. Among the seven included studies with adequate outcome measures on smell function, 76/206 (36.9%) patients underwent reconstruction with a nasoseptal flap. As previously reported, this rate is even smaller in our series (2.4%). In summary and according to Harvey et al., minimising mucosal trauma combined with respecting the olfactory-bearing areas of the nasal cavity is likely to ensure minimal impact on olfaction after pituitary surgery [46]. To avoid damage to the olfactory-bearing septal and turbinate areas, the authors proposed a small, olfactory-preserving nasoseptal flap, the so-called olfactory strip, which led to lower morbidity while maintaining reconstruction options [46].

### Strengths and limitations

To the best of our knowledge, this is the largest single-centre series focusing on objective rhinologic outcomes after ETT surgery and provides a comprehensive update of the current evidence on this topic. Although we aimed to gather available evidence as comprehensively as possible, certain limitations need to be addressed. First, our synopsis reflects the quality of the included studies, which are mostly retrospective case series, involving multiple centres, with study periods covering several decades. Second, the systematic search revealed a large heterogeneity in terms of the reported rhinologic outcomes, including different follow-up algorithms and methods of assessing smell function. Third, because of the heterogeneity of the included studies, no *I*^2^ values could be reported. Fourth, the search via PubMed could have missed certain studies, since other databases (e.g. Embase, Web of Science) were not systematically reviewed.

## Conclusion

Early and late postoperative rhinologic complication rates after ETT surgery for pituitary lesions seem to be low. However, a thorough preoperative evaluation by monorhinal smell testing, in particular in patients at risk for nasoseptal flap harvesting, may optimize long-term rhinologic outcome.

## Supplementary Information

Below is the link to the electronic supplementary material.

**Supplemental Material, Table 1:** Studies included for systematic review are presented with the outcome of interest, for which they met inclusion criteria. EPC; early postoperative complications (epistaxis, sinusitis), LPC; late postoperative complications (synechiae, crusting, septal perforation), NR; not reported, NSF; rate of nasoseptal flaps, SPNF, subjective postoperative nasal function; OPSF; objective postoperative smell function, Y; Yes. (DOCX 16 KB)
