# Supplementary material for: Rhinologic outcome of endoscopic transnasal-transsphenoidal pituitary surgery: an institutional series, systematic review, and meta-analysis
Source: Eur Arch Otorhinolaryngol. 2023 Mar 29;280(9):4091–9. doi: 10.1007/s00405-023-07934-w (PMC10382340; doi:10.1007/s00405-023-07934-w)
Supplement: Supplementary file 1 — Supplemental Material, Table 1: Studies included for systematic review are presented with the outcome of interest, for which they met inclusion criteria. EPC; early postoperative complications (epistaxis, sinusitis), LPC; late postoperative complications (synechiae, crusting, septal perforation), NR; not reported, NSF; rate of nasoseptal flaps, SPNF, subjective postoperative nasal function; OPSF; objective postoperative smell function, Y; Yes. (DOCX 16 KB) [file 405_2023_7934_MOESM1_ESM.docx]

|  | **Number of Pts** | **EPC** | **LPC** | **OPSF** | **SPNF** | **NSF** | **Follow-up** | **Year** |
| --- | --- | --- | --- | --- | --- | --- | --- | --- |
| **Tan et al.** | 25 | Y | Y | NR | Y | NR | NR | 1995 |
| **Jho et al.** | 50 | Y | Y | NT | NR | NR | NR | 1997 |
| **Cappabiancha et al.** | 146 | Y | NR | NR | NR | NR | Y | 2002 |
| **Kabil et al.** | 300 | Y | Y | NR | NR | NR | Y | 2005 |
| **Frank et al.** | 40 | Y | Y | NR | NR | NR | Y | 2006 |
| **Haruna et al.** | 132 | Y | Y | NR | Y | NR | Y | 2006 |
| **Charalampaki et al.** | 150 | Y | NR | NR | Y | NR | Y | 2009 |
| **Graham et al.** | 71 | Y | NR | NR | Y | NR | Y | 2009 |
| **Hart et al.** | 57 | NR | NR | Y | NR | NR | Y | 2010 |
| **Rotenberg et al.** | 17 | NR | Y | Y | NR | 17/17 (100%) | Y | 2011 |
| **Subermann et al.** | 50 | Y | NR | NR | Y | NR | Y | 2011 |
| **Sowerby et al.** | 22 | NR | NR | Y | Y | 4/22 (18.2%) | Y | 2012 |
| **Kahilogullari et al.** | 25 | NR | Y | Y | NR | 25/25 (100%) | Y | 2013 |
| **Alobid et al.** | 36 | NR | NR | Y | Y | 36/36 (100%) | Y | 2013 |
| **Tam et al.** | 20 | NR | NR | Y | NR | 10/20 (50%) | Y | 2013 |
| **Magro et al.** | 300 | Y | Y | NR | Y | 3/300 (1%) | Y | 2016 |
| **Schreiber et al.** | 29 | Y | Y | Y | Y | 5/29 (17%) | Y | 2019 |
| **Baudracco et al.** | 11 | NR | NR | NR | Y | NR | Y | 2020 |
| **Cho et al.** | 52 | NR | NR | NR | Y | NR | Y | 2020 |

**Supplemental Table 1**
